# Validity and reliability of the virtual audit tool for estimating built-environment characteristics in Taiwan

**DOI:** 10.1186/s12942-025-00419-5

**Published:** 2025-10-28

**Authors:** Yi-Chien Yu, Yu-Hsiang Peng, Liang-Rong Chen, Shao-Hsi Chang

**Affiliations:** 1 Department of Physical Education, Tunghai University, Taichung, Taiwan; 2 Taipei Municipal Zhongshan Junior High School, Taipei, Taiwan; 3 Department of Physical Education and Sports Sciences, National Taiwan Normal University, 162, Heping East Road Section 1, Taipei 106, Taipei, Taiwan

**Keywords:** Virtual audit tool, Built environment auditing, Google street view, Street view imagery, Environmental health

## Abstract

**Background:**

Environmental factors significantly influence health behaviors and outcomes. While Google Street View (GSV) has emerged as a cost-effective tool for environmental auditing in various countries, its feasibility in Taiwan remains unexplored. This study aimed to examine the validity and reliability of GSV-based environmental audits in Taiwan.

**Methods:**

Four administrative districts in Taipei representing different population densities and socioeconomic status were selected. A total of 74 street segments within 40 streets were evaluated using both virtual and field audits. The S-VAT was modified to include 8 categories (38 items) of neighborhood characteristics. To assess criterion validity, field and virtual audits were conducted by one rater with a minimum two-week interval. Inter-rater reliability was evaluated by comparing two raters’ virtual audit results, while intra-rater reliability was assessed through repeated virtual audits by the same rater. Cohen’s Kappa and percentage agreement were used for statistical analysis.

**Results:**

Walking-related (k = 0.768), cycling-related (k = 0.921), and public transport features demonstrated high reliability. Lower reliability was found in aesthetics and grocery stores, primarily due to GSV limitations: aesthetic features (litter, graffiti) were affected by viewing angles and temporal variations, while grocery stores were challenging to assess due to restricted storefront visibility and signage clarity.

**Conclusions:**

The S-VAT demonstrates good validity and reliability for environmental auditing in Taiwan, particularly for transportation-related features. However, caution should be exercised when assessing grocery stores and aesthetic features. This study validates GSV as a feasible tool for conducting environmental audits in Taiwan.

## Background

Physical inactivity and food overconsumption are two major risk factors that lead to obesity [1, 2]. Residential neighborhoods may directly or indirectly influence health behaviors including physical activity, sedentary behavior, and dietary consumption [3]. For instance, A study of 8,185 Latin American adults examined the associations between perceived neighborhood built environment characteristics and domain-specific physical activity, finding that higher land use mix–access (OR = 1.27; 95% CI: 1.13–1.43), greater land use mix–diversity (OR = 1.12; 95% CI: 1.05–1.20), more walking and cycling facilities (OR = 1.18; 95% CI: 1.09–1.28), and better aesthetics (OR = 1.10; 95% CI: 1.02–1.18) were all positively associated with engaging in at least 10 min of leisure-time physical activity per week [4]. A review of 13 studies from seven countries examined the relationship between neighborhood walkability and weight-related behaviors or outcomes among children and adolescents (*n* = 98 to 37,460), findings showed that eight studies (61.5%) reported higher level of walkability was associated with active lifestyles and healthy weight status [5]. Consequently, there are growing studies that have examined how environmental factors of neighborhood walkability (connectivity, land-use mix, population density) contribute to active living and health. It is significant to explore the environmental factors associated with health in order to establish policies and design appropriate behavioral change programs.

Previous studies have commonly employed field audits to collect data on environmental characteristics. Traditional in-field auditing methods for assessing the built environment often necessitate that investigators undergo specialized training as auditors. However, these approaches are resource-intensive, time-consuming, and prone to inconsistencies arising from variations in auditors’ perceptions and behaviors [6]. Moreover, field observations may lead to participant discomfort or ethical concerns, especially when researchers are visibly present in residential areas [7]. These limitations have highlighted the need for more scalable and less intrusive methods for assessing neighborhood environments, such as virtual audit tools.

In the last decade, there has been a growing number of studies using Google Street View (GSV) as a tool to evaluate environmental attributes. Compared to traditional field audits, GSV-based approaches reduce the demand for time and cost, while increasing scalability and standardization across study sites [8]. Recent studies have demonstrated GSV’s applicability in various national and cross-national settings. For instance, over 164 million GSV images from across the United States have been analyzed using computer vision models to quantify walkability and other environmental features [8]. Likewise, another large-scale study extracted built environment indicators from approximately 31 million GSV images across 7.8 million intersections using computer vision models to examine associations with health behaviors and outcomes at the census tract level [9]. In addition, a systematic review of 96 studies employing street view imagery (SVI) found that 89 studies (92.7%) used GSV for built environment assessments, highlighting its widespread application in this field [10]. Given the growing evidence supporting GSV-based audits and the limitations of traditional field approaches, it is essential to explore the feasibility and applicability of such tools in other geographic and cultural contexts. However, it is still unknown whether GSV is feasible to be used in Taiwan.

The SPOTLIGHT Virtual Audit Tool (S-VAT) was developed by Lakerveld et al. [11] to assess the obesogenicity of neighborhoods. The validity (vs. field audit) and reliability (using test-retest) of the tool was previously demonstrated in a random sample of streets from four Dutch neighborhood [12]. S-VAT presents several advantages making it suitable for validation in diverse countries. (1) It is developed based on validated tools, drawing from previous virtual and field audit instruments. This ensures that the S-VAT is built upon a reliable foundation. (2) the S-VAT incorporates a standardized operating procedure, detailing how each item should be scored. This reduces inter-rater variability and enhances the accuracy of the results. (3) the S-VAT has demonstrated validated reliability and validity, with studies showing moderate-to-high inter-rater and intra-rater reliability, as well as validity when compared with field audit^[12]^. Moreover, the S-VAT is suitable for cross-national comparisons due to its standardized design and application in multiple European cities [13–16]. A recent systematic review highlighted that the S-VAT standardized virtual audit tools, enhances the comparability and generalizability of research findings across diverse environments [10]. This underscores the importance of cultural adaptability in audit instruments to ensure accurate interpretation of built environment features in different contexts. In summary, although environmental characteristics may vary across countries, the structured format of the S-VAT, its clearly defined scoring criteria, and its visual-based assessment approach support its potential adaptability to local conditions. These strengths suggest that the S-VAT may serve as a promising tool for evaluating the applicability of GSV-based audits beyond its original European context.

Building upon prior evidence demonstrating the effectiveness of GSV and the utility of the S-VAT, this study aimed to examine the feasibility of using GSV for environmental auditing in Taiwan. The S-VAT was employed to assess the validity and reliability of GSV-based environmental audits in the Taiwanese context to filling the gap in evidence for its use in Asian urban environments.

## Methods

### Sampling

**Fig. 1:**
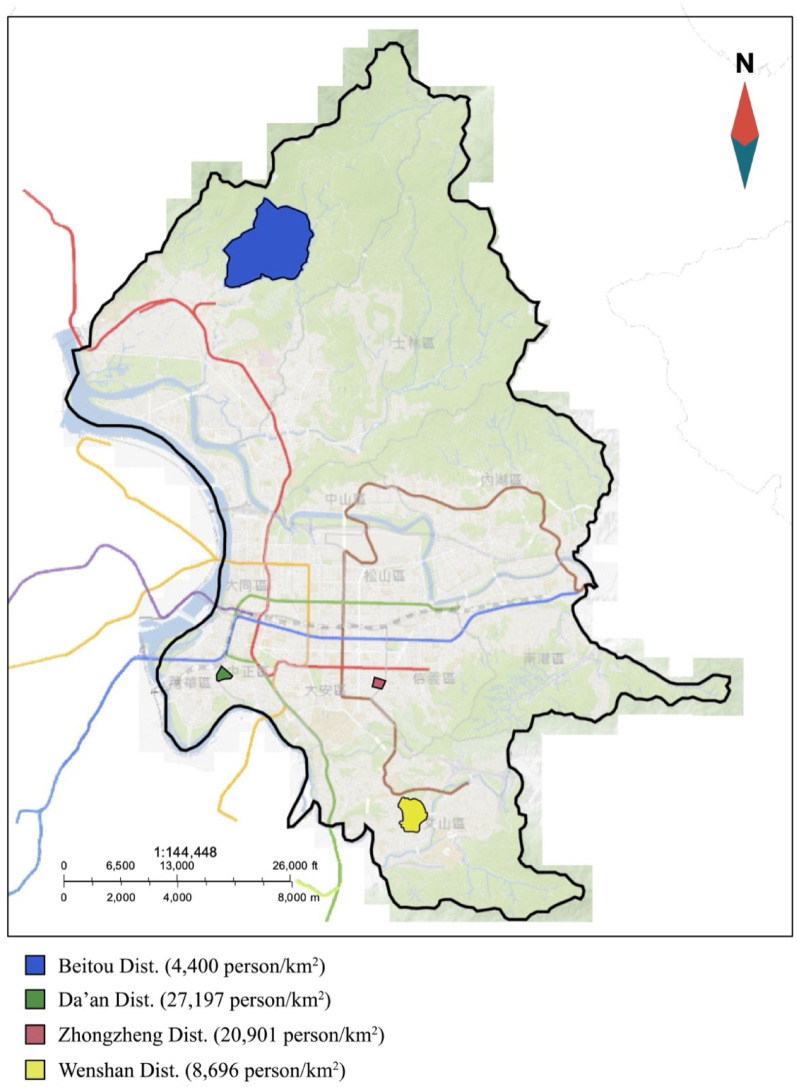
Study area (Four administrative districts in Taipei City were randomly selected for validity and reliability testing of the Short-form Virtual Audit Tool (S-VAT): Da’an (27,197 persons/km²), Zhongzheng (20,901 persons/km²), Wenshan (8,696 persons/km²), and Beitou (4,400 persons/km²)

This study conducted the virtual and field audit of neighborhoods in April 2021. This study randomly selected four administrative units in Taipei, the capital of Taiwan, were selected to test the validity and reliability of the S-VAT. These units comprised Da’an (population density: 27,197 persons/km²), Zhongzheng (20,901 persons/km²), Wenshan (8,696 persons/km²), and Beitou (4,400 persons/km²) districts (Fig. 1), representing variations in population density and socioeconomic status (SES). These districts demonstrated a clear population density gradient, ranging from highly urbanized Da’an district to the relatively less densely populated Beitou district. Population density was calculated using population counts and neighborhood area data from the Department of Civil Affairs, Taipei City Government [17, 18]. The household median income data across neighborhoods were obtained from the Fiscal Information Agency, Ministry of Finance [19]. We categorized these neighborhoods into tertiles according to population density and SES. The first and third tertiles were extracted to represent low population density/household income and high population density/household income, respectively. The four neighborhood categories (i.e., high population density-high SES, high population density-low SES, low population density-high SES, low population density-low SES) were created using cross tabulation. One neighborhood in each group was randomly selected because of feasibility. Street segments were defined as the part of the street between two intersections, with a length of 50–300 m; shorter segments (< 50 m) were merged if they belonged to a continuous street. If streets crossed neighborhood boundaries, they were audited either entirely or extended up to 300 additional meters when the continuation exceeded the boundary [12].

### The Google street View-based virtual audit tool

**Fig. 2:**
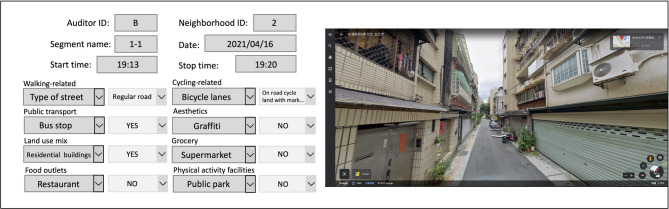
Data input form alongside Google Street View. (The audit form was designed to be completed while viewing Google Street View in a split-screen layout. On the left, the standardized observation form captures environmental features. On the right, the corresponding Google Street View image of the audited street segment is displayed, enabling synchronized assessment of physical and social environmental attributes.)

The SPOTLIGHT Virtual Audit Tool (S-VAT) was modified for this study to better suit Taiwan’s urban context. Three items were removed from the original S-VAT due to their subjective nature and poor fit with local conditions. These included take away restaurants (difficulty in exterior assessment), condition ratings of residential buildings (subjective maintenance evaluation), and residential versus non-residential building ratios (incompatibility with Taiwan’s prevalent mixed-use architecture). This modification approach aligns with previous studies that have adapted S-VAT categories to address geographical and cultural considerations or specific target populations [20]. The final version of S-VAT tool incorporated 8 categories (38 items) of neighborhoods characteristics: walking-related items, cycling-related items, public transport, aesthetics, land use-mix, grocery stores, food outlets, and recreational facilities. Each of the categories included separate factors (e.g., type of street and sidewalk condition) as displayed in Table 1. Each factor was rated using one of three measurement scales: binary (yes/no), ordinal (e.g., poor/fair/good), or continuous (counting the number of specific built environment features). A data input form was created with drop-down menu options for all responses. The form was designed to be viewed alongside Google Street View using a computer split screen (Fig. 2).

**Table 1:** Percentage agreement and kappa statistics of all categories

Category	Criterion validity		Inter rater reliability		Intra rater reliability	
	Percentage agreement	kappa	%	kappa	%	kappa
Walking-related*	**97.1**	**0.855**	**90.7**	**0.768**	**100**	**1.000**
Type of street	98.6	0.976	94.6	0.907	100	1.000
Pedestrian friendly street						
Traffic sharing road						
Regular road						
Road with high-speed traffic						
Sidewalk binary (Yes/No)	98.6	0.973	97.3	0.946	100	1.000
Sidewalk Good Fair Poor Under construction	97.3	0.947	97.3	0.946	100	1.000
Pedestrian binary (Yes/No)	97.3	0.490	97.3	0.946	100	1.000
Zebra path (Yes/No)	94.6	0.887	68.9	0.027	100	1.000
Over underpass (Yes/No)	98.6	0.000	90.5	0.797	100	1.000
Traffic lights (Yes/No)	93.2	0.862	100	1.000	100	1.000
Streetlight (Yes/No)	98.6	0.850	79.7	0.582	100	1.000
Cycling-related*	**96.2**	**0.936**	**95.4**	**0.921**	**100**	**1.000**
Type of bicycle lanes binary (Yes/No)	95.9	0.000	100	1.000	100	1.000
Type of bicycle lanes On road cycle lane with markings Shared path with pedestrians Separate cycle lane with buffer	73.0	0.255	97.3	0.934	100	1.000
Obstacles present No obstacle Temporary Permanent	100	1.000	100	1.000	100	1.000
Traffic calming devices (Yes/No)	90.5	0.317	91.9	0.527	100	1.000
Public bicycle facilities (Yes/No)	97.3	0.490	97.3	0.930	100	1.000
Public transport*	**99.3**	**0.920**	**98.0**	**0.718**	**99.3**	**0.920**
Bus stop (Yes/No)	98.6	0.916	95.9	0.707	98.6	0.916
MRT station (Yes/No)	100	1.000	100	1.000	100	1.000
Aesthetics*	**83.4**	**0.592**	**86.5**	**0.609**	**96.6**	**0.912**
Tree (Yes/No)	86.5	0.731	85.1	0.701	100	1.000
Graffiti (Yes/No)	90.5	0.716	81.1	0.349	93.2	0.820
Litter (Yes/No)	64.9	0.235	87.8	0.144	94.6	0.747
Abandoned vacant area (Yes/No)	93.2	0.723	91.9	0.583	97.3	0.884
Land use mix*	**93.7**	**0.862**	**84.2**	**0.658**	**98.6**	**0.970**
Residential buildings visible (Yes/No)	100	1.000	95.9	0.387	100	1.000
Type of residential (Yes/No) buildings binary	100	1.000	94.6	0.308	100	1.000
5below	87.8	0.740	71.6	0.363	98.6	0.967
5up	93.2	0.831	91.9	0.788	98.6	0.966
Detached Detached semidetached homes No	94.6	0.874	77.0	0.374	97.3	0.938
Terraced Terraced homes No	86.5	0.732	74.3	0.460	97.3	0.944
Grocery stores*	**96.5**	**0.802**	**90.0**	**0.430**	**98.9**	**0.940**
Supermarket (Yes/No)	98.6	0.794	97.3	0.491	98.6	0.794
Local food shop (Yes/No)	93.2	0.815	74.3	0.494	100	1.000
Street food market (Yes/No)	95.9	0.240	86.5	0.056	98.6	0.796
Wine liquor store (Yes/No)	97.3	0.815	93.2	0.000	98.6	0.883
Convenience store (Yes/No) small grocery store (Yes/No)	97.3	0.886	90.5	0.604	98.6	0.940
Food outlets*	**97.0**	**0.842**	**92.2**	**0.577**	**99.2**	**0.958**
Restaurant (Yes/No)	87.8	0.781	73.0	0.484	95.9	0.929
Fast food restaurant (Yes/No)	100	1.000	100	1.000	100	1.000
Café Bar (Yes/No)	97.3	0.880	93.2	0.683	100	1.000
On street vendors of food (Yes/No)	100	1.000	94.7	0.323	100	1.000
Shopping mall (Yes/No)	100	1.000	100	1.000	100	1.000
Physical activity facilities*	**99.5**	**0.939**	**97.3**	**0.587**	**100**	**1.000**
Indoor recreational facilities (Yes/No)	100	1.000	98.6	0.793	100	1.000
Outdoor facilities (Yes/No)	100	1.000	97.2	0.000	100	1.000
Public Park (Yes/No)	98.6	0.882	95.9	0.606	100	1.000
Overall**	**95.3**	**0.844**	**91.8**	**0.659**	**99.1**	**0.963**

Bold text indicates the main categories

*Mean results of Percentage Agreement and Kappa for reliability and validity tests per category

**Mean results of Percentage Agreement and Kappa for reliability and validity tests summarized for all categories

### Procedure

To test the criterion validity of the S-VAT, the first rater (YC) conducted the field audit by traveling to the selected street segments, both sides, in person on 40 streets, with a minimum interval of two weeks before conducting virtual audits in a reversed order on the same streets using the S-VAT. This approach aimed to minimize potential bias by preventing the researcher from being influenced by previous experiences of auditing the same street segments. To assess the virtual audit’s inter-rater reliability, the two raters’ (YC and YX) records of the same street segments from virtual audits were compared. Before the virtual audit was conducted, the assessors received training on how to use the S-VAT within the street view feature of GE. The same two raters then conducted the virtual audit. To ensure the virtual audit’s intra-rater reliability the same rater (YC) conducted two virtual audits to evaluate the intra-observer variability. In order to minimize the potential impact of recall bias, a minimum of two weeks elapsed between the two audits for each street. This design was adopted to rigorously examine both the reliability and validity of the virtual audit method. Criterion validity was assessed to verify whether S-VAT could produce results comparable to traditional field-based assessments. Inter- and intra-rater reliability testing aimed to ensure the tool’s consistency across different raters and over time—key indicators of its robustness and reproducibility in practice. This approach was also consistent with the validation procedures adopted in the original development of the SPOTLIGHT Virtual Audit Tool, which employed the same three strategies—inter-rater reliability, intra-rater reliability, and criterion validity—to establish its measurement properties [12].

**Fig. 3:**
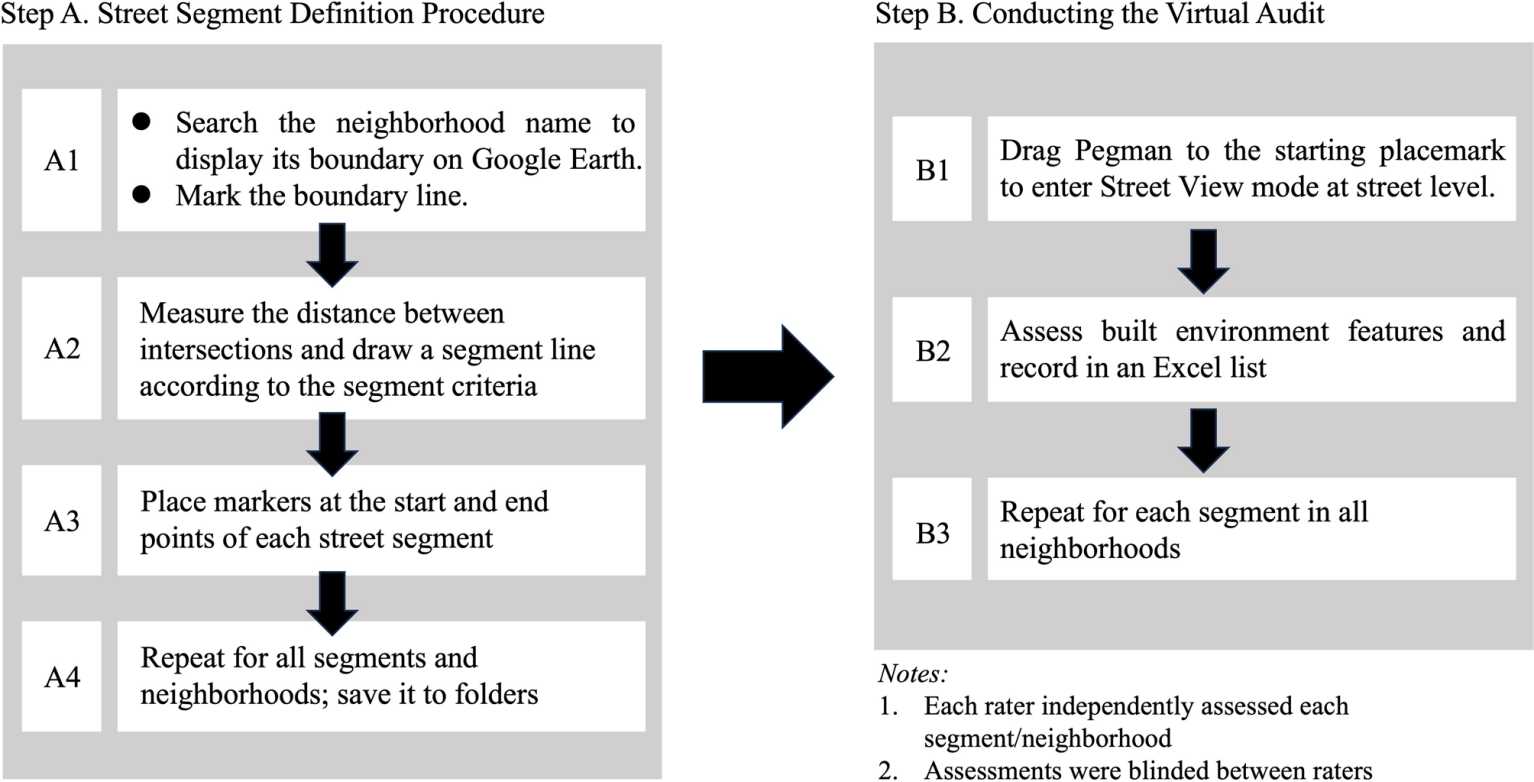
Virtual Audit Procedure. (The figure illustrates two main steps for conducting the Short-form Virtual Audit Tool (S-VAT). Step A describes how street segments were defined using Google Earth, including boundary identification, distance measurement, and placement of segment markers. Step B shows the virtual audit process, including navigation in Google Street View, assessment of built environment features, and recording in an Excel sheet. Notes indicate that each rater independently assessed all neighborhoods and that assessments were blinded between raters.)

The procedures for conducting the virtual audit are illustrated in Fig. 3. The time taken by both the field and virtual audits was recorded. The assessments of the two raters were blinded from each other. The field audits were conducted during daytime on weekdays.

### Analysis

Cohen’s Kappa is a statistical coefficient that measures inter-rater agreement for categorical items. Unlike simple percent agreement, it accounts for the agreement occurring by chance [21]. It is calculated as: *Po =* Observed agreement = the proportion of instances where both raters agree. *Pe =* Expected agreement by chance = the proportion of agreement expected if both raters were assigning categories randomly but maintaining their marginal proportions.

$$\:k=\frac{Po-Pe}{1-Pe}$$

Kappa coefficient values are commonly interpreted as follows: < 0.20 (poor agreement), 0.21–0.40 (fair), 0.41–0.60 (moderate), 0.61–0.80 (substantial), and 0.81–1.00 (almost perfect agreement) [21]. In this study, Cohen’s Kappa (κ) was used to assess three dimensions of agreement: (1) inter-rater reliability (i.e., consistency between different raters), (2) intra-rater reliability (i.e., consistency of the same rater over time), and (3) criterion validity (i.e., agreement between virtual and field audits). These comparisons allowed us to evaluate the consistency of observations across raters, within raters, and between audit modes. Moreover, Kappa is a standardized reliability metric widely adopted in virtual audit studies across countries [12, 22].

In addition to Kappa, we reported the percentage of agreement to provide a supplementary descriptive measure, particularly in cases where high agreement may be obscured by low Kappa values due to skewed marginal distributions. All analyses were performed using Microsoft Excel (version 16.6) and SPSS (version 23.0).

## Results

A total of 74 street segments within 40 streets were evaluated for agreement between virtual and field audits. Table 1 describes the percentage agreement and kappa statistics of each item across eight categories. Rater A (YC) had an average remoting observation from 3.76 to 4.23 min/street and 3.35 min/route field audit. The online ratings by rater B (YX) average time of 5.43 min/street. The results showed moderate to almost perfect inter- and intra-rater agreement over the majority of street characteristic categories (Inter-rater reliability: 91.8%; Intra-rater reliability: 99.1%). Other, almost perfect agreement was reported between field audits and virtual audits by using GSV.

Intra-rater reliability showed a high average between the first and second virtual audit, ranging from 96.6% agreement (k = 0.91) to 100% agreement (k = 1.00). Moreover, the reliability result was high in four categories (walking-related, cycling-related, food outlets, and physical activity facilities) for both agreement (100%) and kappa values (k = 1.00).

Inter-rater reliability results were high (average 92.5%) for the majority of street characteristics, however, in a specific category (grocery stores, food outlets, and physical activity facilities) were moderate Kappa values (k = 0.430; 0.577; 0.587).

Percentage agreement between field and virtual audit was high across all street characteristic categories with results ranging from 83.4 to 99.5% agreement (average 91.4%) and the kappa coefficient ranging from k = 0.592 to 0.942 (moderate to almost perfect agreement).

(Percentage agreement and kappa statistics are reported for 38 items grouped into eight categories of neighborhood characteristics: walking-related items, cycling-related items, public transport, aesthetics, land use-mix, grocery stores, food outlets, and recreational facilities. Values marked with an asterisk indicate mean results per category; double asterisks indicate mean results summarized across all categories.)

## Discussion

This study evaluated the validity and reliability of the S-VAT to assess the physical environmental characteristics in Taiwan. While our results demonstrated high reliability in assessing walking-related (k = 0.768), cycling-related (k = 0.921), and public transport features (k = 0.718), lower inter-rater reliability was observed in aesthetics (k = 0.609), grocery stores (k = 0.430), and physical activity facilities (k = 0.587), suggesting potential challenges in assessing these environmental characteristics through virtual audits.

The category of grocery stores displayed a high percentage of agreement (90.0%), but moderate kappa values (k = 0.430). This discrepancy may be attributed to the different mathematical properties of these statistical measures. Percent agreement serves as an absolute measure of agreement, while kappa statistics account for chance agreement [23]. Although percentage agreement can be reliable when auditors are well-trained and items are dichotomous, these results warrant careful interpretation.

The challenges in grocery store assessment emerged from two main aspects. First, raters faced difficulties in understanding store categories when store signs were not clearly interpretable, leading to more guesswork in their assessment. This challenge was particularly pronounced when stores were closed, making it difficult to determine the store category. Second, different street views could be observed when accessing images from different entry points of cross intersections, potentially introducing bias in the assessment. For instance, we observed two streets where night markets operated, causing the streetscape to vary significantly between day and night. When entering these streets from different positions (side A or B of the street), completely different street views were observed. Previous studies had indicated that when virtually crossing intersections, such inconsistencies were frequently observed [24], resulting in temporal inconsistencies in the year or season of images used for audits [25]. These findings suggest that the grocery store characteristics in the original S-VAT tool may need modifications to better reflect Taiwan’s retail environment. Future studies should consider developing locally adapted criteria for assessing grocery stores in Taiwan.

The overall aesthetic assessment in this study demonstrated moderate to high reliability and validity (criterion validity: k = 0.592; inter-rater reliability: k = 0.609; intra-rater reliability: k = 0.912), which differs from the relatively lower aesthetic assessment results reported by Bethlehem et al. [12] (intra-rater: k = 0.654; inter-rater: k = 0.440; criterion validity: k = 0.539). However, consistent with Bethlehem et al. [12] findings, both studies revealed lower kappa values for items related to litter (criterion validity: k = 0.235; inter-rater reliability: k = 0.144) and graffiti (inter-rater reliability: k = 0.349). The lower agreement in litter assessment could be attributed to the varying street views at different entry points of cross intersections, similar to the challenges encountered in grocery store assessment. Different entrances of crossing intersections might result in different street views, potentially causing raters to observe different aspects of litter presence. Additionally, the assessment of graffiti requires a higher degree of subjective judgment, which may explain the relatively lower inter-rater reliability in this category. Some previous studies have pointed to the aesthetics category being subjected to smaller nuances and subjective judgment [26, 27]. Therefore, the challenges in GSV-specific viewing angle constraints may affecting litter assessment, while graffiti assessment is influenced by raters’ subjective interpretation. Future research could explore standardized viewing angle protocols for GSV-based assessment and develop more structured evaluation criteria for graffiti to minimize subjective variations in environmental audits.

While the S-VAT tool is designed to assess a wide range of indicators in residential neighborhoods, certain categories may be rarely observed or inapplicable in different cultural contexts. These variations could be attributed to distinct urban development patterns between Asian and Western countries. For instance, Taiwan cities typically feature higher housing density, with detached houses predominantly located in rural or suburban areas. Thus, using a common type of Asian house could potentially have allowed determining the accurate agreement in the land-use mix category, despite the agreement having been reported at a substantial level.

The limitations of GSV-based virtual audits in capturing building heights represent another methodological challenge. GSV images, captured by car-mounted cameras, effectively provide street-level perspectives but may have limited capability in assessing vertical dimensions [22]. In contrast, field auditing allows raters to adjust their viewing positions, enabling more comprehensive assessment of building characteristics. This limitation suggests the need for complementary methods or technological solutions for accurate building height assessment in virtual audits.

There are several limitations to consider. First, the study site was conducted in the capital of Taiwan. Although this study referred to previous designs that used different types of neighborhoods, further increased the representativeness of the study. However, rural streets may lack or have an outdated image compared to urban areas [28]. As a result, generalizing results to other areas (such as suburban or rural areas) should be done with caution, since further region validation is required. Second, GSV images may have some specific temporal validity issues [24, 29], which is a common problem reported in most GSV tools [1, 30]. For example, on-street litters or construction work signage are fickle and may or might not be present at the time when the GSV car drives through. In addition, even though we confirmed that the evaluated Google Street View (GSV) images were from the same year, it cannot be overlooked that these images may have been updated during the evaluation process. Consequently, these might have affected the inter-rater reliability. Finally, although the inter-rater reliability results demonstrated acceptable agreement in this study, the involvement of only two raters limits the generalizability and robustness of the reliability estimates, as variability among a larger group of raters could not be assessed.

## Conclusion

The S-VAT appears to be a generally valid and reliable evaluation of the environmental characteristics associated with physical activity and dietary behaviors in Taipei, Taiwan. However, the tool should be applied with caution in categories of land use mix, aesthetic and grocery stores in Taiwan. This study also demonstrates the applicability of the S-VAT virtual audi

t tool in an Asian urban context, addressing the gap left by previous research conducted primarily in European settings. These findings suggest that S-VAT can serve as a feasible option for built environment assessment in diverse cultural regions. Future studies are encouraged to expand its application to other Asian cities to strengthen cross-cultural research contributions.

## Data Availability

No datasets were generated or analysed during the current study.

## Abbreviations

GSV: Google Street View
S-VAT: Spotlight Virtual Audit Tool
SVI: Street View Imagery
SES: Socioeconomic Status
